# Progressive gray matter reduction in schizophrenia patients with persistent auditory hallucinations by causal structural covariance network analysis

**DOI:** 10.1017/S0033291725101438

**Published:** 2025-08-29

**Authors:** Xu Shao, Honghong Ren, Lulin Dai, Jingqi He, Jinguang Li, Ying He, Xiangzhen Kong, Xiaogang Chen, Jinsong Tang

**Affiliations:** 1 Department of Psychiatry, Zhejiang University School of Medicine, Sir Run Run Shaw Hospital, Zhejiang, China; 2 Hunan Provincial Brain Hospital, Hunan, China; 3 Department of Clinical Psychology, Shandong Provincial Hospital Affiliated to Shandong First Medical University, Shandong, China; 4 Department of Psychiatry, The Second Xiangya Hospital of Central South University, Hunan, China; 5 Department of Psychology and Behavioral Sciences, Zhejiang University, Zhejiang, China; 6 Zigong Mental Health Center, Sichuan, China

**Keywords:** auditory hallucination, causal structural covariance network, gray matter volume, schizophrenia, thalamus

## Abstract

**Background:**

Schizophrenia patients with auditory hallucinations have distinct morphological abnormalities, but whether this population have a progressive gray matter atrophy pattern and specific transmission chain of causal effects remains unclear. This study was designed to construct a causal structural covariance network in schizophrenia patients with persistent auditory hallucinations.

**Methods:**

T1-weighted MRI images were acquired from 90 schizophrenia patients with persistent auditory hallucinations (pAH group) and 83 healthy controls (HC group). Stage-specific independent *t* tests of gray matter volume (GMV) comparisons between the two groups were used to depict the GMV atrophic pattern and locate the atrophic origin. In the pAH group, the causal structural covariance network (CaSCN) was constructed to map causal effects between the atrophic origin and other regions as the auditory hallucination severity increased.

**Results:**

With the ascending of hallucinatory severity, GMV reductions began from the thalamus, bilateral medial frontal gyri, left Rolandic operculum, and left calcarine, and expanded to other frontal and temporal regions, hippocampal complex, insula, anterior cingulate gyri, fusiform, and cerebellum. Using the peak region (thalamus) as the causal origin in the network, transitional nodes including the right opercular part of the inferior frontal gyrus, bilateral postcentral gyri, left thalamus, and right middle frontal gyrus received the casual information and projected to target nodes from the frontal, temporal, parietal, and occipital cortices, limbic system, and cerebellum.

**Conclusions:**

Our study revealed causal effects from the thalamus and a specific transmission pattern of causal information within the network, indicating a thalamic–cortical–cerebellar circuitry dysfunction related to auditory hallucinations.

## Introduction

Auditory hallucinations are sensory experiences that individuals perceive voices as distinct from their own thoughts without external stimuli (American Psychiatric Association, 2013). They are debilitating psychotic symptoms affecting 60–80% of patients with schizophrenia (Lim, Hoek, Deen, & Blom, 2016). Auditory hallucinations often contain negative content, including personal insult and command of violent behavior (Larøi et al., 2012), which brings severe damage to patients’ mental condition, including depressive symptoms and suicidal ideation (Chiang, Beckstead, Lo, & Yang, 2018; Yin et al., 2023). Despite no consensus on the definition of persistent auditory hallucinations, they can be defined as persistent symptoms for more than one year after treatment with two different antipsychotic drugs (González, Aguilar, Berenguer, Leal, & Sanjuan, 2006). After adequate pharmaceutical treatment, over a quarter of patients with schizophrenia still suffer from auditory hallucinations (Shergill, Murray, & McGuire, 1998).

Abundant neuroimaging studies have revealed that schizophrenia patients with auditory hallucinations had distinct morphological abnormalities compared to non-hallucinatory patients. Voxel-based morphometry studies have focused mainly on the gray matter volume (GMV), and patients with auditory verbal hallucinations exhibited increased gray matter volumes in the right Heschl’s gyrus, and reduced volumes in the left insula, right temporal pole, and cerebellum lobule VIII (Amad et al., 2014; Cierpka et al., 2017; Ren, Li, Zhou, et al., 2024a; Shapleske et al., 2002). Meta-analyses revealed that the severity of auditory hallucinations was negatively correlated with GMV in the left insula and bilateral superior temporal gyri (Modinos et al., 2013; Palaniyappan, Balain, Radua, & Liddle, 2012). Although these studies revealed the association between the gray matter atrophy and auditory hallucinations, results were inconsistent. Heterogeneous findings implicate that auditory hallucinations may have intricate pathogenesis, and simple cross-sectional regional studies may be not able to fully depict the pathological pattern. On the other hand, the heterogeneity may be caused by that patients from different studies underwent different phases of the illness or different severity levels of auditory hallucinations. Therefore, performing morphological analyses with the information of illness duration or symptom severity taken into consideration may help reveal neuroanatomical substrates of auditory hallucinations.

The conventional structural covariance network measures the synchronization of interregional morphological changes and cannot reflect the temporal progression of morphological abnormalities (Prasad et al., 2022). Comparatively, the causal structural covariance network (CaSCN) analysis is an effective way of portraying the progressive gray matter profiles of structural networks (Zhang et al., 2017). This analysis is able to establish causal relationships among regions regarding gray matter changes, and capture the origin of causal relationships in the network. In detail, preprocessed gray matter images of a group are first rearranged in an ascending order based on the illness duration or symptom severity to create pseudo-time series. Then, the Granger causality analysis (GCA) is applied to the sequenced images to construct a directed network. The GCA is widely used to identify causal relationships between time series and characterize the brain temporal information flow in fMRI studies (Zang, Yan, Dong, Huang, & Zang, 2012). The CaSCN analysis combines the structural covariance network and the GCA techniques to calculate directed connectivity values among regions for quantifying the causal effects of gray matter volume changes. This analysis has gained popularity in neuroimaging studies on both somatic and psychiatric diseases, including the Parkinson’s disease, posttraumatic stress disorder, and major depressive disorder (Chen et al., 2022; Li et al., 2022; Lu et al., 2023).

So far, CaSCN studies on schizophrenia are lacking. Jiang et al. (2018) revealed that with the increase of illness duration, the gray matter atrophy in patients with schizophrenia began in the thalamus and progressed to the frontal cortex, temporal cortex, occipital cortex, and cerebellum (Jiang et al., 2018). However, no studies have been conducted on schizophrenia patients with auditory hallucinations. Whether this specific population had a unique progressive gray matter atrophy pattern and transmission chain of causal effects remains unclear. Hence, this study was designed to construct a causal structural covariance network using 3D T1-weighted MRI images of schizophrenia patients with persistent auditory hallucinations and age- and sex-matched healthy controls. We first examined the stage-specific progressive pattern of gray matter volumes in schizophrenia patients with auditory hallucinations based on the severity of auditory hallucinations. Then, we constructed the whole-brain voxel-wise and region-of-interest (ROI)-wise CaSCNs to evaluate causal effects between the atrophic origin and other brain regions. We hypothesized that schizophrenia patients with persistent auditory hallucinations would exhibit a progressive gray matter atrophy pattern with the increase of hallucinatory severity.

## Method

### Participants

In total, 90 schizophrenia patients with persistent auditory hallucinations (pAH) were enrolled from the psychiatric clinic of the Second Xiangya Hospital of Central South University in China, and 83 age- and sex-matched healthy controls (HC group) were enrolled from local communities (Ren, Li, Li, et al., 2024b; Shao et al., 2024). All patients were diagnosed according to the Diagnostic and Statistical Manual of Mental Disorders, Fifth Edition (DSM-5) by two experienced psychiatrists and all participants were assessed via the Chinese version of the Mini-International Neuropsychiatric Interview (M.I.N.I.) (Sheehan et al., 1998). The inclusion criteria for all participants were as follows: (1) Han Chinese descent and aged between 16 and 45 years; (2) right-handed; and (3) normal hearing. The additional inclusion criteria for schizophrenia were: (1) experiences of auditory verbal hallucinations at least once daily for at least one year; (2) unresponsiveness to at least two types of antipsychotics; and (3) a score >3 on PANSS P3 item (Psomiades et al., 2018; Ren et al., 2022; Wang et al., 2022). Based on Andreasen et al., 2005, P3 ≤ 3 is set as the threshold for symptomatic remission (Andreasen et al., 2005). The exclusion criteria for all participants were as follows: (1) a history of other psychiatric disorders; (2) a history of drug or alcohol abuse; (3) a history of head trauma with consciousness disturbances lasting more than 5 min; (4) a history of severe endocrine or other physical illnesses; (5) pregnant or lactating; and (6) MRI incompatibility. The study was conducted in accordance with the Declaration of Helsinki and was approved by the Ethics Committee of Zhejiang University School of Medicine Sir Run Run Shaw Hospital (No. lunshen2022yandi0081). Each participant provided a written informed consent and had the right to withdraw during the study procedure.

### Clinical measurements

The Positive and Negative Syndrome Scale (PANSS) is used to evaluate the severity of psychiatric symptoms (Kay, Fiszbein, & Opler, 1987). This measure has three subscales, including the Positive Scale (POS), Negative Scale (NEG), and General Psychopathology Scale (GPS). In accordance with previous studies, the P3 hallucination item in the PANSS was used to evaluate the severity of auditory hallucinations (Andreasen et al., 2005; Benetti et al., 2015).

The Psychotic Symptom Rating Scales (PSYRATS) is a commonly used scale to assess various dimensions of auditory hallucinations and delusions (Haddock, McCarron, Tarrier, & Faragher, 1999). This measure has two subscales including the auditory hallucinations subscale (AHS) and delusions subscale (DS). A higher score indicates more severe symptoms. The AHS score was also used to measure the severity of auditory hallucinations in our study.

### MRI acquisition

After enrollment, all participants received an MRI scan via a 3.0T MRI scanner (Siemens, Munich, Germany) with a 16-channel head coil at the Magnetic Imaging Center of Hunan Children’s Hospital, Changsha, China. T1-weighted MRI data were acquired via a 3D magnetization prepared rapid acquisition gradient echo (3D MPRAGE) sequence with the following parameters: repetition time = 2530 ms, time to echo = 2.33 ms, field of view = 256 × 256 mm, flip angle = 7°, slice thickness = 1 mm, 192 sagittal slices, and voxel size = 1 mm^3^. There was no major scanner upgrade or instrument replacement during the study period. During the scanning, foam pads were used to restrain head movement and earplugs to attenuate noise. Participants were instructed to lie on their back and keep their head and body motionless.

### Image preprocessing

The original images were first visually inspected to determine any distortion or motion artifact, and no participant was excluded because of the raw image quality. T1-weighted images were then preprocessed via the New-segment and DARTEL function in the DPABI toolbox (Data Processing Analysis of Brain Imaging toolbox, http://rfmri.org/dpabi) (Chao-Gan & Yu-Feng, 2010). Raw images were normalized to the Montreal Neurologic Institute (MNI) space, segmented into gray matter, white matter, and cerebrospinal fluid, resampled to 1.5 × 1.5 × 1.5 mm^3^, and modulated to acquire gray matter volume images. The preprocessed images were smoothed by a Gaussian kernel filter with a 6-mm full width at half-maximum (FWHM) and visually checked. Three participants in the pAH group were excluded because of poor segmentation.

### Stage-specific GMV alteration patterns in the pAH group

In the pAH group, participants were first subgrouped into four groups using the quartiles’ approach based on the PSYRATS AHS score to ensure a proximate population distribution (stage 1, 14–24 scores; stage 2, 25–29 scores; stage 3, 30–32 scores; and stage 4, 33–42 scores). In each subgroup, the independent *t* test was used to compare the difference of gray matter volumes between patients and healthy controls with age, sex, years of education, and total intracranial volume (TIV) as covariates. *P* < 0.00001 after false discovery rate (FDR) correction was considered statistically significant (Jiang et al., 2018). To ensure that group differences were not caused by an arbitrary grouping strategy, patients were also subgrouped into three groups according to the PANSS P3 score (stage 1, P3 = 4; stage 2, P3 = 5; and stage 3, P3 = 6 or 7) (only one participant scored 7 on the PANSS P3 item) for replication.

### Voxel-wise CaSCN construction

The GCA technique applied in the CaSCN was originally employed in temporal series to detect causal effects. Given time courses X and Y, if the current value of Y could be better predicted by the past values of X and Y than the past value of Y itself, it can be inferred that X should have a Granger causal effect on Y (Granger, 1969). The CaSCN analysis applies the GCA on the pseudo-time series through sequencing GMV images in a specified order to calculate the causal effect of one brain region atrophy on another.

In detail, all gray matter volume images in our patient groups were sequenced from low to high by the ranks of PYRATS AHS score and PANSS P3 score. This created a pseudo-time series for portraying GMV alterations on cross-sectional data. The brain region showing the most significant GMV reduction (maximal |*t* value|) compared with healthy controls at the first stage was considered as the origin of the progressive gray matter atrophy pattern, and was selected as the seed region for the CaSCN construction. The signed path coefficient GCA was conducted via the REST-GCA toolkit implemented in REST1.8 software (http://www.restfmri.net) by a voxel-wise manner using a gray matter mask (Zang et al., 2012). Sex, age, years of education, TIV, illness duration, onset age, and chlorpromazine equivalent dosage were set as covariates. Under the circumstance of the seed region showing GMV reductions, a positive Granger causality (GC) value of a region represents that GMV reductions of this region lags behind the seed region, suggesting the reduction is driven by the seed (Zhang et al., 2017). To acquire statistically meaningful GC effects, the GC map was *z* transformed and corrected by the GRF method (*z* > 3.29 and corresponding GC value >0.43) (Li et al., 2022).

### ROI-wise CaSCN construction

To conduct the ROI-wise network analysis, ROIs were extracted from the GC map earlier to investigate the causal link between the seed region and other regions. The signed path coefficient GCA was conducted on these ROIs to investigate GC relationships among regions (Zhang et al., 2017). Resultant positive GC values were preserved and transformed by the *z* distribution, with a *p* < 0.05 representing a significant GC value (Li et al., 2022; Xu et al., 2023). Then, the weighted out-degree, in-degree, and net-degree of each ROI were calculated. For each ROI, an out-degree stands for the sum of signed path coefficients from this node projecting to other nodes; an in-degree stands for the sum of signed path coefficients from other nodes projecting to this node; and a net-degree results from subtracting in-degree from out-degree and stands for the total causal effect of this node in the network. A positive net-degree shows a causal source in the network, while a negative one shows a causal target in the network (Jiang et al., 2018; Xu et al., 2023).

## Results

### Demographic and clinical features

The chi-square test revealed that there was no difference in the sex distribution between the pAH group and the HC group (*χ* ^2^ = 0.08, *p* = 0.77). Independent samples *t* tests revealed that there was no age difference between two groups (*t* = 1.76, *p* = 0.08), and the pAH group had fewer years of education than the HC group (*t* = 6.16, *p* < 0.001) (Table 1). The clinical information of the pAH group was also shown in Table 1.

**Table 1.** Sociodemographic features and clinical information of all participants

	pAH group (*n* = 87)	HC group (*n* = 83)
Sex (M/F)	40/47	40/43
Age (year)	25.24 ± 5.58	26.80 ± 5.91
Education (year)	11.70 ± 3.12*	14.43 ± 2.65
Onset age (year)	18.46 ± 4.89	
Duration (year)	6.85 ± 4.41	
First treatment age (year)	19.15 ± 5.22	
CPZ (mg/day)	675.89 ± 337.46	
PANSS P3	5.08 ± 0.74	
PANSS POS	16.24 ± 4.16	
PANSS NEG	15.21 ± 5.48	
PANSS GPS	27.44 ± 6.85	
PANSS total	59.13 ± 13.52	
PSYRATS AHS	28.41 ± 6.03	
PSYRATS DS	11.57 ± 7.38	
PSYRATS total	39.99 ± 10.88	

*Note:* PANSS P3, the P3 item of PANSS; PANSS POS, the Positive Scale of PANSS; PANSS NEG, the Negative Scale of PANSS; PANSS GPS, the General Psychopathology Scale of PANSS; PSYRATS AHS, the auditory hallucinations subscale of PSYRATS; PSYRATS DS, the delusions subscale of PSYRATS. *p < 0.05 compared with the HC group.

Abbreviations: M, male; F, female; CPZ, chlorpromazine equivalent dosage.

### Stage-specific gray matter volume analysis

**Figure 1.**
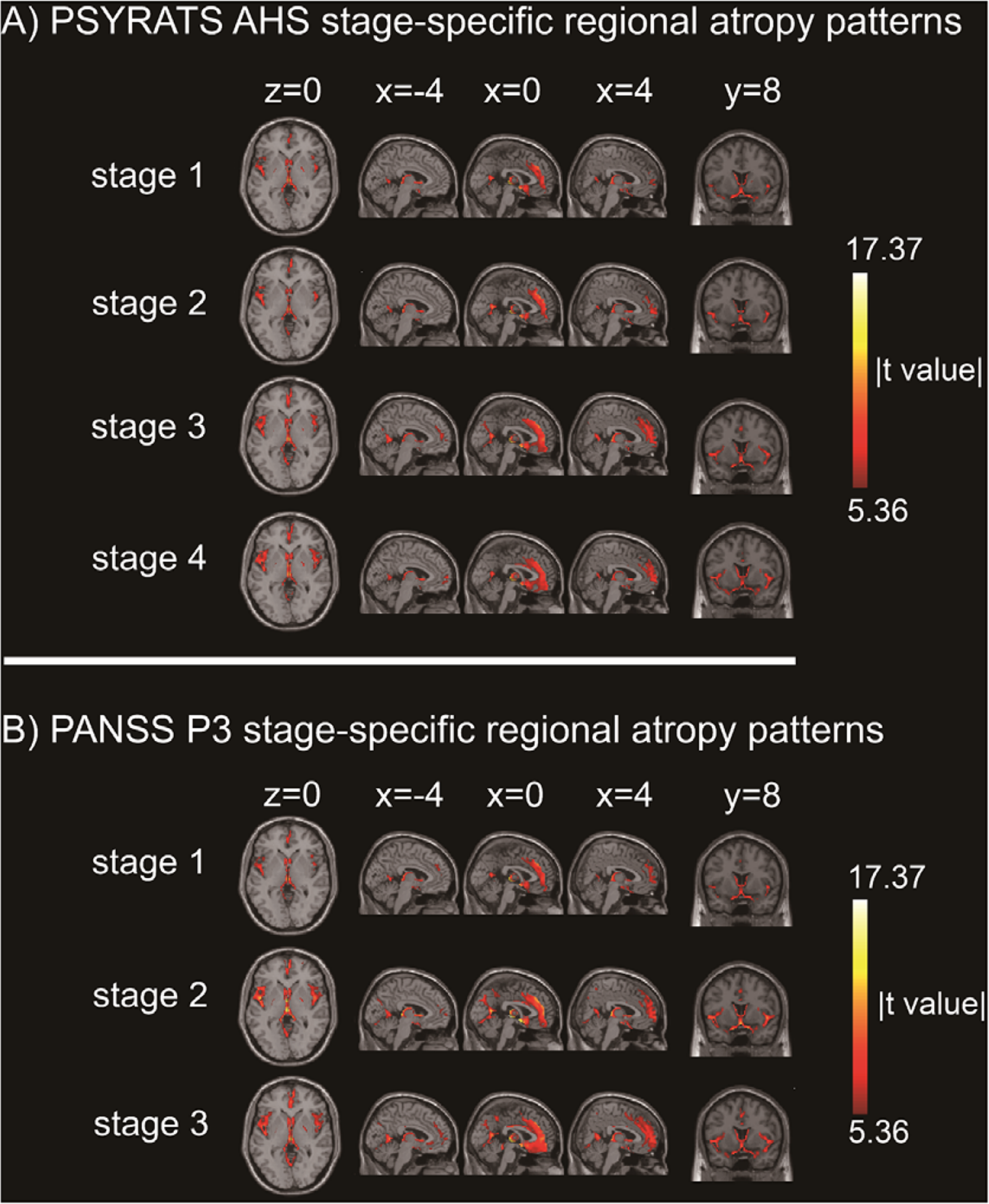
Stage-specific progressive pattern of gray matter atrophy in schizophrenia patients with persistent auditory hallucinations. Color bar represents *t* values from independent samples *t* tests between patient subgroups and healthy controls. (a) The pAH group was divided into four subgroups according to the PSYRATS AHS score (stage 1/2/3/4 = 14–24/25–29/30–32/33–42); (b) The pAH group was divided into three subgroups according to the PANSS P3 item score (stage 1/2/3 = 4/5/6–7).

To depict progressive GMV changes with an increased severity of auditory hallucinations in the pAH group, patients were first divided into four groups using the quartiles’ approach based on the PSYRATS AHS score (stage 1/2/3/4 = 14–24/25–29/30–32/33–42). The chi-square test and ANOVA, respectively, showed that there were no differences among four subgroups regarding sex distribution (*χ* ^2^ = 1.02, *p* = 0.80) and age (*F* = 0.84, *p* = 0.48) (Supplementary Table S1). Stage-specific *t* tests showed that there were progressive atrophy patterns with a greater severity of auditory hallucinations. As shown in Figure 1a, at stage 1 (*N* = 21, 11 men, mean age 24.95 ± 6.77 years), four clusters exhibited reduced GMVs in the pAH group, including the thalamus (mainly extending to the bilateral hippocampal complex) (peak coordinate: 0, −23, 2; *t* = −13.39), bilateral medial frontal gyri (mainly extending to the bilateral anterior cingulate gyri) (peak coordinate: 0, 41, 32; *t* = −10.19), left Rolandic operculum (mainly extending to the left superior temporal gyrus and temporal pole) (peak coordinate: −45, −6, 3; *t* = −8.82), and left calcarine (peak coordinate: 0, −59, 9; *t* = −9.49). At stage 2 (*N* = 24, 11 men, mean age 25.54 ± 5.80 years), atrophic regions at stage 1 continued to extend. Aside from that, the cerebellum exhibited more extensive GMV reduction at this stage. At stage 3 (*N* = 23, 11 men, mean age 24.00 ± 4.15 years), clusters from the thalamus and medial frontal gyrus merged into one single atrophic cluster. Aside from that, the inferior and middle frontal gyri exhibited more extensive GMV reduction. At stage 4 (*N* = 19, 7 men, mean age 26.68 ± 5.44 years), the GMV atrophy continued with atrophic areas from the frontal cortex, temporal cortex, thalamus, hippocampus, and cerebellum merging into a bigger cluster. See Supplementary Table S2 in detail. Then, patients were divided into three groups according to the PANSS P3 item score (stage 1/2/3 = 4/5/6–7). Since only one participant scored 7 on the P3 item, those who scored either 6 or 7 were divided into the same group. Our study revealed that there were no significances among groups regarding sex distribution (*χ* ^2^ = 4.64, *p* = 0.20) and age (*F* = 1.13, *p* = 0.34) (Supplementary Table S3). Similar alteration patterns were observed in this categorized strategy (Figure 1b). See Supplementary Table S4 in detail. Notably, no GMV increase was found in either categorized strategies.

Independent samples *t* tests showed that among regions exhibiting reduced GMVs in stage 1, the thalamus-centered cluster had the greatest statistical significance (FDR corrected *p* < 10^−5^). The GMV reduction in the peak region of the thalamus was still significant after setting the threshold to even higher levels (until FWE corrected *p* < 10^−9^), indicating that the GMV atrophy in this region was the most severe at stage 1 (Supplementary Figure S1).

### Voxel-wise CaSCN analysis

**Figure 2.**
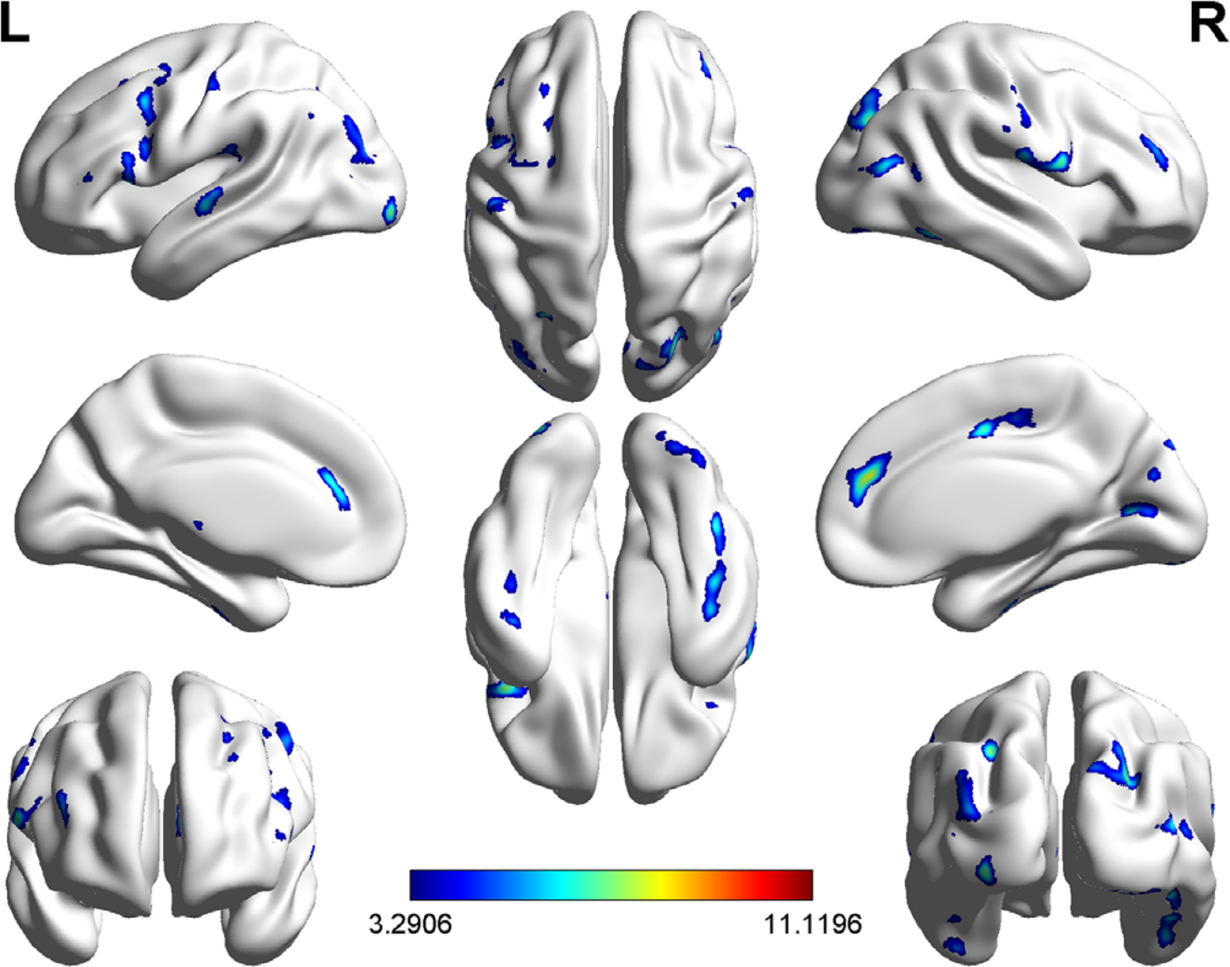
The voxel-wise causal structural covariance network in schizophrenia patients with persistent auditory hallucinations. The causal network was constructed by applying Granger causality analysis to sequenced morphometric images according to the ascending order of hallucinatory severity. The thalamus (0, −23, 2) was utilized as the seed region. GC values were *z* transformed and corrected by the GRF method (*z* > 3.29).

The peak region of the thalamus with a maximal absolute *t* value during *t* tests at stage 1 was selected as the seed region of the voxel-wise CaSCN analysis (peak coordinate: 0, −23, 2; *t* = −13.39) with the seed radius set as 6 mm. The CaSCN analysis showed that the thalamus had positive causal effects in multiple regions, indicating that these regions had reduced GMVs after the GMV reduction of the peak region, including the bilateral middle frontal gyri, right opercular part of the inferior frontal gyrus, left triangular part of the inferior frontal gyrus, right Rolandic operculum, bilateral superior temporal gyri, right middle temporal gyrus, left inferior temporal gyrus, bilateral postcentral gyri, left inferior parietal lobule, left supramarginal gyrus, bilateral angular gyri, right precuneus, right calcarine, bilateral middle occipital gyri, left inferior occipital gyrus, right fusiform gyrus, bilateral anterior cingulate gyri, right medial cingulate gyrus, left thalamus, right cerebellum lobule II, left cerebellum lobules IV–V, and left cerebellum lobule VIII (Table 2; Figure 2).

**Table 2.** Brain regions showing causal effect from the seed of thalamus by using causal structural covariance network analysis

Region	MNI coordinate			GC value	*Z*	Voxel	
	x	y	z			peak	cluster
Frontal_Mid_L	−25.5	15	46.5	0.70	5.35	107	116
Frontal_Mid_L	−27	33	40.5	0.64	4.88	89	100
Frontal_Mid_R	40.5	45	22.5	0.78	5.96	317	319
Frontal_Inf_Oper_R	48	15	0	0.86	6.55	113	240
Frontal_Inf_Tri_L	−45	16.5	6	0.99	7.54	387	1687
Frontal_Inf_Tri_L	−45	34.5	4.5	0.63	4.79	127	127
Rolandic_Oper_R	57	3	15	0.96	7.35	283	541
Temporal_Sup_L	−63	−19.5	1.5	0.88	6.71	192	412
Temporal_Sup_R	46.5	−43.5	22.5	0.77	5.87	44	137
Temporal_Mid_R	46.5	−52.5	7.5	1.14	8.65	223	275
Temporal_Inf_L	−40.5	−9	−34.5	1.03	7.84	389	397
Postcentral_L	−49.5	−16.5	46.5	0.73	5.57	322	322
Postcentral_R	31.5	−36	46.5	0.83	6.33	82	100
Postcentral_R	60	−10.5	39	0.82	6.24	517	763
Parietal_Inf_L	−28.5	−63	42	1.00	7.63	67	187
Supramarginal_L	−49.5	−25.5	22.5	0.75	5.70	150	599
Angular_L	−42	−57	33	0.98	7.48	212	217
Angular_R	42	−58.5	30	0.83	6.36	102	102
Precuneus_R	15	−61.5	37.5	1.38	10.48	277	1418
Calcarine_R	19.5	−69	9	0.95	7.21	547	547
Occipital_Mid_L	−37.5	−84	19.5	0.72	5.51	721	755
Occipital_Mid_R	39	−73.5	13.5	1.00	7.62	194	443
Occipital_Inf_L	−27	−91.5	−6	1.23	9.38	139	297
Fusiform_R	42	−15	−28.5	1.06	8.08	409	1205
Cingulum_Ant_L	−9	33	21	0.90	6.83	266	318
Cingulum_Ant_R	7.5	43.5	22.5	1.07	8.13	443	555
Cingulum_Mid_R	9	−6	42	0.92	7.02	450	680
Thalamus_L	−6	−22.5	4.5	0.99	7.53	242	253
Cerebelum_Crus2_R	7.5	−85.5	−30	1.46	11.12	1807	7562
Cerebelum_4_5_L	−12	−42	−19.5	0.56	4.30	243	316
Cerebelum_8_L	−39	−51	−49.5	0.76	5.81	457	589

*Note:* Abbreviations of brain regions were according to the AAL template. L, left; R, right. *Z* values were corrected by GRF method, *p* < 0.05.

### ROI-wise CaSCN analysis

**Figure 3.**
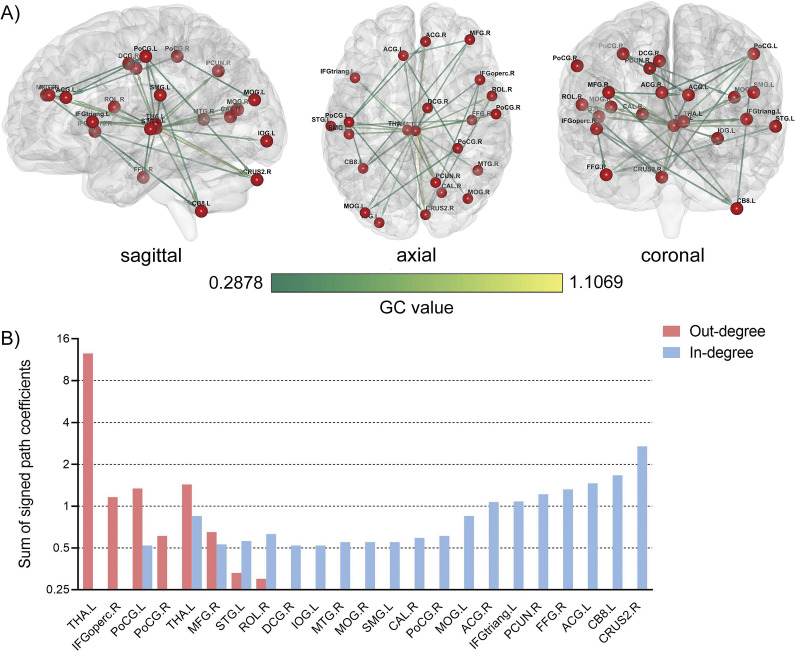
The ROI-wise causal structural covariance network in schizophrenia patients with persistent auditory hallucinations. (a) Signed path coefficient-based ROI-wise CaSCN with the arrow direction pointing to the recipient of causal information flow; (b) Weighted out-degree, in-degree, and net-degree of each ROI in the network. A positive net-degree shows a causal source in the network, while a negative one shows a causal target in the network. Regions were ranked according to the descending order of the net degree.

The seed region and nodes derived from the voxel-wise CaSCN analysis were further utilized to conduct a ROI-wise CaSCN analysis. Positive GC values were preserved and *z* transformed, and the weighted out-degree, in-degree, and net-degree of each ROI were calculated. Our study found positive net-degrees in the right opercular part of the inferior frontal gyrus, bilateral postcentral gyri, left thalamus, and right middle frontal gyrus, indicating these regions may be transitional nodes in the directed network receiving casual information from the seed region of the thalamus. Our study found negative net-degrees in the left triangular part of the inferior frontal gyrus, left superior temporal gyrus, right middle temporal gyrus, right Rolandic operculum, right medial cingulate gyrus, bilateral anterior cingulate gyri, left inferior occipital gyrus, bilateral middle occipital gyri, left supramarginal gyrus, right calcarine, right postcentral gyrus, right precuneus, right fusiform, left cerebellum lobule VIII, and right cerebellum lobule II, indicating that these regions may be target nodes in the directed network receiving the causal information from the seed region via transitional nodes (Supplementary Table S5; Figure 3).

## Discussion

To the best of our knowledge, our study was the first to apply the casual structural covariance network analysis in schizophrenia patients with persistent auditory hallucinations, and investigate casual relationships among regions with progressive morphological changes. Stage-specific gray matter volume analyses revealed that along with the increase of hallucinatory severity, the thalamus, bilateral medial frontal gyri, left Rolandic operculum, and left calcarine had reduced gray matter volume at an early stage, and gradually expanded to other frontal and temporal cortices, hippocampal complex, insula, anterior cingulate gyri, fusiform, and cerebellum. Using the peak region (thalamus) as the causal seed, the voxel-wise CaSCN demonstrated positive granger causalities from the seed to regions including middle and inferior frontal gyri, Rolandic operculum, superior, middle, and inferior temporal gyri, postcentral gyrus, inferior parietal lobule, supramarginal gyrus, angular gyrus, precuneus, calcarine, middle and inferior occipital gyri, fusiform, anterior and medial cingulate gyri, left thalamus, and cerebellum. The ROI-wise CaSCN analysis revealed that transitional nodes including the right opercular part of the inferior frontal gyrus, bilateral postcentral gyri, left thalamus, and right middle frontal gyrus received the casual information from the seed region of the thalamus, and projected to target nodes including regions from the frontal cortex, temporal cortex, parietal cortex, occipital cortex, limbic system, and cerebellum.

To date, only one study has applied the causal structural covariance network in patients with schizophrenia. Constructing pseudo-time series according to the illness duration, Jiang et al. (2018) found that the thalamus showed gray matter reductions at an early stage, and served as the primary hub of the causal network. The frontal regions, as transitional points, received causal effects from the thalamus, and projected to temporal cortices, occipital cortices, and cerebellum (Jiang et al., 2018). In accordance with the study by Jiang et al. (2018), our study also found the thalamus as the seed region of the causal network. The thalamus is considered as a diencephalic structure composed of heterogeneous nuclei, and has distinct synaptic input and cortical connection (Giraldo-Chica & Woodward, 2017). It is primarily responsible for the transmission of sensory input such as early-stage visual or auditory stimuli, and contributes to the integration of cognitive processing including attention function, executive function, and memory (Halassa & Kastner, 2017; Wolff & Vann, 2019). Previous studies have found that patients with schizophrenia had volume reductions in the thalamus (Brickman et al., 2004; Byne et al., 2001), deficient cortical–thalamic correlation patterns (Mitelman et al., 2005), and reduced functional activation (Yamamoto et al., 2018), indicating that morphological and functional abnormalities of the thalamus play a crucial role in the pathogenesis of schizophrenia. Besides, the thalamus is also associated with the mechanism of auditory hallucinations. According to the forward model of auditory hallucinations, the sensory motor system responds to the voice generated by the motor command and develops an actual sensory feedback, while an efference copy of motor command is used for sensory feedback prediction. The predicted auditory feedback often matches the actual feedback when the sound is self-generated, then the sensory input is suppressed, leading to a dampened auditory experience. By contrast, the predicted auditory feedback contradicts with the actual feedback when the sound is externally produced, leading to no suppression of an auditory experience (Heinks-Maldonado et al., 2007; Wolpert & Miall, 1996). In this model, the thalamus is responsible for transmitting the prediction error to the premotor area and involves in the interaction between the sensory feedback and higher level cognition (Pinheiro, Schwartze, & Kotz, 2020). The dysfunction of any link during the forward model may induce the occurrence of auditory hallucinations. Thus, in view of the role of thalamus in this cognitive model, the gray matter atrophy of thalamus may have a negligible impact on forward model deficits.

Our study found that transitional nodes in the causal network of schizophrenia patients with persistent auditory hallucinations included the right opercular part of the inferior frontal gyrus, bilateral postcentral gyri, left thalamus, and right middle frontal gyrus, which differed from those in the causal network of schizophrenia constructed by Jiang et al. (2018). Despite of the similar causal origin between the two networks, the hallucinatory network may have a distinct transitional pattern of causal effects. Previous neuroimaging studies have supported these regions were associated with auditory hallucinations. The meta-analysis of task-state fMRI studies revealed that the activation of the right inferior frontal gyrus was specifically related to auditory verbal hallucinations (van Lutterveld, Diederen, Koops, Begemann, & Sommer, 2013). A PET study revealed that schizophrenia patients with auditory verbal hallucinations had increased metabolic activities in the right middle frontal gyrus (Kopecek et al., 2007). An fMRI study discovered reduced effective connectivity from the anteromedial prefrontal cortex to the right middle frontal gyrus in schizophrenia patients with auditory verbal hallucinations (Zhao et al., 2018). Compared to healthy controls, schizophrenia patients had increased functional connectivity from the thalamus to multiple sensory–motor regions including the bilateral postcentral gyri (Ferri et al., 2018). The meta-analysis of auditory verbal hallucinations also revealed that the presence of auditory verbal hallucinations was related to the activation in the bilateral postcentral gyri (Kühn & Gallinat, 2012). Based on these evidences, functional activities in the right inferior frontal gyrus, right middle frontal gyrus, and bilateral postcentral gyri were more active in hallucinatory patients, supporting that these node regions play a significant role in transmitting causal effects in the directed network related to auditory hallucinations.

Target nodes of the directed network constructed in our study included frontal, temporal, occipital, parietal, limbic, and cerebellar regions, indicating that the gray matter volume of the thalamus had extensive causal effects on the whole brain. Among all the regions influenced by the causal origin, cerebellar regions including the left cerebellum lobule VIII and right cerebellum lobule II had the lowest net-degree values, indicating these regions were mostly modulated by the causal effect from the thalamus. In schizophrenia patients with persistent auditory verbal hallucinations, a morphological study found gray matter volume reduction in the cerebellum lobule VIII and a negative relationship between the gray matter volume of the cerebellum lobule VIII and overall positive symptoms (Cierpka et al., 2017). An fMRI study revealed that schizophrenia patients with auditory verbal hallucinations had weaker functional connectivity between the bilateral medial pulvinar nucleus and cerebellum and stronger effective connectivity from the left medial pulvinar nucleus to the right inferior frontal gyrus, indicating an aberrant association between the cerebellar–thalamic–cortical circuit and auditory verbal hallucinations (Wei et al., 2022). In addition, impaired cerebellar function is linked to the prediction error of sensory feedback in the forward model, and thus induces auditory hallucinations and higher level cognitive dysfunction (Pinheiro et al., 2020). Therefore, structural abnormalities of the cerebellar subfields were highly associated with auditory hallucinations.

However, our study still has some shortcomings. First, our study was a cross-sectional neuroimaging study, and a longitudinal follow-up study could have depicted a clearer picture of the atrophic pattern of the whole brain gray matter volume with the increase of auditory hallucination severity. Second, our study focused on the causal effect from the thalamus to other brain regions in the structural level, and an additional investigation in the functional level could have reflected the causal influence from the thalamus in the auditory hallucination network more comprehensively. Third, the thalamus is composed of a group of heterogeneous nuclei, and a study on the gray matter atrophy and causal effects of different thalamic subfields could have revealed more specific causal effects of the thalamus. Finally, the causal structural covariance network was constructed using schizophrenia patients with persistent auditory hallucinations and healthy controls, and a methodological improvement allowing comparisons among three or more groups would help reveal more distinct structural features related to auditory hallucinations.

Insights emerging from mapping intrinsic brain connectivity networks provide a potentially mechanistic framework for an understanding of human behaviors including auditory hallucinations (Cheng et al., 2025; Mo et al., 2024; Zhang, Xu, Ma, Qian, & Zhu, 2024). Information flow from quantifying causal effects in the directed brain networks has a strong potential to enhance this framework. Our study was the first to apply the causal structural covariance network in schizophrenia patients with persistent auditory hallucinations and construct a directed network of auditory hallucinations based on gray matter volumes. This study has revealed causal effects of the thalamus and its specific transmission pattern within the network, showing a thalamic–cortical–cerebellar circuit of morphological alterations. The causal network has provided hints for investigating the pathogenesis of auditory hallucinations from a structural network level and finding tailored neuromodulation treatment with a potential brain circuitry target.

## Supporting information

Shao et al. supplementary material Shao et al. supplementary material

## Supplementary material

The supplementary material for this article can be found at http://doi.org/10.1017/S0033291725101438.
