# Supplementary material for: Progressive gray matter reduction in schizophrenia patients with persistent auditory hallucinations by causal structural covariance network analysis
Source: Psychol Med. 2025 Aug 29;55:e255. doi: 10.1017/S0033291725101438 (PMC13040588; doi:10.1017/S0033291725101438)
Supplement: Shao et al. supplementary material [file S0033291725101438sup001.docx]

**Supplementary Table 1.** Demographic and clinical characteristic of pAH subgroups divided according to the PSYRATS AHS score

|  | **Stage 1** (n=21) | **Stage 2** (n=24) | **Stage 3** (n=23) | **Stage 4**  (n=19) | **HC** (n=83) | **Statistic** | ***p*** |
| --- | --- | --- | --- | --- | --- | --- | --- |
| Age (year) | 24.95±6.77 | 25.54±5.80 | 24.00±4.15 | 26.68±5.44 | 26.80±5.91 | 1.37 | 0.25 |
| Sex (M/F) | 11/10 | 11/13 | 11/12 | 7/12 | 40/43 | 1.10 | 0.89 |
| Education (year) | 12.67±3.38 | 11.29±3.01 | 11.20±2.53 | 11.74±3.56 | 14.43±2.65a,b,c,d | 10.38 | **<0.001** |
| Onset age (year) | 19.38±5.32 | 17.50±5.47 | 17.30±2.65 | 20.05±5.43 |  | 1.69 | 0.17 |
| Disease duration  (year) | 5.67±3.73 | 8.08±5.29 | 6.65±3.87 | 6.84±4.43 |  | 1.15 | 0.33 |
| First treatment age  (year) | 19.86±5.62 | 18.00±5.70 | 18.17±2.99 | 21.00±5.96 |  | 1.61 | 0.19 |
| CPZ (mg/d) | 703.77±362.23 | 678.61±273.15 | 630.40±372.65 | 696.71±359.46 |  | 0.21 | 0.89 |
| PANSS P3 | 4.67±0.66 | 5.04±0.55 | 5.30±0.82a | 5.32±0.75a |  | 3.98 | **0.01** |
| PANSS total | 52.67±11.84 | 60.46±11.64 | 62.64±14.78 | 60.53±14.53 |  | 2.34 | 0.08 |
| PSYRATS AHS | 20.05±3.22 | 27.33±1.52a | 30.96±0.71a,b | 35.95±2.66a,b,c |  | 189.33 | **<0.001** |
| PSYRATS total | 29.95±8.82 | 37.88±8.05a | 42.09±7.51a | 51.21±8.07a,b,c |  | 23.91 | **<0.001** |

**Note:** The PSYRAT AHS ranges from 14 to 24 at stage 1, from 25 to 29 at stage 2, from 30 to 32 at stage 3, and from 33 to 42 at stage 4. pAH, schizophrenia with persistent auditory hallucinations; HC, healthy control; M, male; F, female; CPZ, Chlorpromazine equivalent dosage; PANSS, the Positive and Negative Syndrome Scale; PSYRATS, the Psychotic Symptom Rating Scales; AHS, the auditory hallucinations subscale of PSYRATS. The statistic values were obtained by chi-square test or ANOVA. a, *p*<0.05 compared to patients at stage 1; b, *p*<0.05 compared to patients at stage 2; c, *p*<0.05 compared to patients at stage 3; d, *p*<0.05 compared to patients at stage 4.

**Supplementary Table 2.** Results of group comparison of GMV between the pAH subgroups and HC group divided according to the PSYRATS AHS score

|  |  | MNI coordinate | | |  | Voxel | |
| --- | --- | --- | --- | --- | --- | --- | --- |
| Cluster | Region | x | y | z | *t* value | region | cluster |
| ***Stage 1*** |  |  |  |  |  |  |  |
| Cluster 1 | Thalamus* | 0 | -22.5 | 1.5 | -13.39 | 250 | 4868 |
| Cluster 1 | ParaHippocampal_R | 13.5 | -4.5 | -18 | -12.87 | 186 | 4868 |
| Cluster 1 | Hippocampus_R | 22.5 | -21 | -13.5 | -12.20 | 259 | 4868 |
| Cluster 1 | Hippocampus_L | -13.5 | -3 | -16.5 | -11.75 | 247 | 4868 |
| Cluster 2 | Frontal_Sup_Medial_L* | 0 | 40.5 | 31.5 | -10.19 | 526 | 1608 |
| Cluster 2 | Cingulum_Ant_L | 0 | 48 | 4.5 | -8.97 | 424 | 1608 |
| Cluster 2 | Frontal_Sup_Medial_R | 3 | 52.5 | 21 | -7.73 | 254 | 1608 |
| Cluster 2 | Cingulum_Ant_R | 1.5 | 54 | 10.5 | -7.53 | 126 | 1608 |
| Cluster 2 | Frontal_Med_Orb_R | 1.5 | 55.5 | -7.5 | -7.11 | 103 | 1608 |
| Cluster 3 | Calcarine_L* | 0 | -58.5 | 9 | -9.49 | 94 | 308 |
| Cluster 3 | Vermis_4_5 | -1.5 | -61.5 | 0 | -6.22 | 40 | 308 |
| Cluster 4 | Rolandic_Oper_L* | -45 | -6 | 3 | -8.82 | 155 | 1075 |
| Cluster 4 | Temporal_Sup_L | -48 | 0 | 0 | -8.55 | 148 | 1075 |
| Cluster 4 | Temporal_Pole_Sup_L | -34.5 | 10.5 | -22.5 | -8.24 | 364 | 1075 |
| Cluster 4 | Frontal_Inf_Orb_L | -43.5 | 16.5 | -3 | -7.92 | 60 | 1075 |
| Cluster 4 | Frontal_Inf_Tri_L | -51 | 16.5 | 0 | -6.56 | 26 | 1075 |
| ***Stage 2*** |  |  |  |  |  |  |  |
| Cluster 1 | Thalamus* | 0 | -22.5 | 1.5 | -13.41 | 183 | 2489 |
| Cluster 1 | Hippocampus_R | 22.5 | -21 | -13.5 | -12.39 | 177 | 2489 |
| Cluster 1 | ParaHippocampal_R | 13.5 | -3 | -18 | -11.94 | 83 | 2489 |
| Cluster 1 | Hippocampus_L | -13.5 | -3 | -16.5 | -10.17 | 160 | 2489 |
| Cluster 2 | Frontal_Sup_Medial_L* | 0 | 33 | 37.5 | -10.48 | 687 | 2107 |
| Cluster 2 | Frontal_Med_Orb_R | 3 | 57 | 0 | -9.34 | 160 | 2107 |
| Cluster 2 | Cingulum_Ant_L | 1.5 | 37.5 | 21 | -8.09 | 403 | 2107 |
| Cluster 2 | Frontal_Sup_Medial_R | 3 | 55.5 | 13.5 | -8.05 | 410 | 2107 |
| Cluster 2 | Frontal_Med_Orb_L | 3 | 63 | -10.5 | -7.10 | 52 | 2107 |
| Cluster 3 | Calcarine_L* | -1.5 | -58.5 | 9 | -10.43 | 96 | 371 |
| Cluster 3 | Vermis_4_5 | 0 | -63 | -1.5 | -7.38 | 84 | 371 |
| Cluster 4 | Rolandic_Oper_L* | -48 | 1.5 | 0 | -9.42 | 160 | 1128 |
| Cluster 4 | Frontal_Inf_Orb_L | -40.5 | 18 | -9 | -8.39 | 82 | 1128 |
| Cluster 4 | Temporal_Pole_Sup_L | -40.5 | 12 | -19.5 | -8.39 | 306 | 1128 |
| Cluster 4 | Frontal_Inf_Tri_L | -52.5 | 18 | 1.5 | -7.77 | 33 | 1128 |
| Cluster 5 | Insula_R* | 33 | -10.5 | 12 | -9.22 | 71 | 121 |
| Cluster 6 | Insula_R* | 43.5 | 19.5 | -6 | -9.14 | 602 | 1070 |
| Cluster 6 | Temporal_Pole_Sup_R | 40.5 | 13.5 | -19.5 | -8.33 | 183 | 1070 |
| Cluster 6 | Rolandic_Oper_R | 46.5 | -10.5 | 9 | -8.25 | 96 | 1070 |
| Cluster 6 | Frontal_Inf_Oper_R | 51 | 16.5 | -1.5 | -6.24 | 11 | 1070 |
| Cluster 7 | Cerebellum_4_5_L* | -25.5 | -27 | -31.5 | -8.58 | 37 | 108 |
| Cluster 7 | Fusiform_L | -24 | -10.5 | -40.5 | -7.55 | 19 | 108 |
| ***Stage 3*** |  |  |  |  |  |  |  |
| Cluster 1 | Thalamus* | 1.5 | -22.5 | 1.5 | -13.17 | 273 | 10031 |
| Cluster 1 | ParaHippocampal_R | 13.5 | -4.5 | -18 | -11.01 | 161 | 10031 |
| Cluster 1 | Frontal_Sup_Medial_L | 0 | 39 | 30 | -10.95 | 810 | 10031 |
| Cluster 1 | Hippocampus_R | 24 | -21 | -13.5 | -10.46 | 215 | 10031 |
| Cluster 1 | Cingulum_Ant_L | 0 | 48 | 4.5 | -10.37 | 769 | 10031 |
| Cluster 1 | Hippocampus_L | -12 | -7.5 | -19.5 | -10.04 | 214 | 10031 |
| Cluster 2 | Frontal_Inf_Orb_L* | -40.5 | 19.5 | -6 | -9.47 | 102 | 1783 |
| Cluster 2 | Rolandic_Oper_L | -46.5 | 1.5 | 0 | -9.04 | 160 | 1783 |
| Cluster 2 | Temporal_Pole_Sup_L | -51 | 10.5 | -3 | -8.88 | 241 | 1783 |
| Cluster 2 | Insula_L | -45 | 6 | -3 | -8.88 | 795 | 1783 |
| Cluster 2 | Frontal_Inf_Tri_L | -51 | 18 | 1.5 | -7.90 | 96 | 1783 |
| Cluster 3 | Cerebellum_3_R* | 19.5 | -27 | -27 | -9.30 | 55 | 106 |
| Cluster 3 | Cerebelum_4_5_R | 24 | -27 | -30 | -8.87 | 36 | 106 |
| Cluster 4 | Frontal_Mid_L* | -24 | 46.5 | 28.5 | -6.89 | 153 | 172 |
| Cluster 4 | Frontal_Sup_L | -25.5 | 51 | 24 | -6.70 | 19 | 172 |
| ***Stage 4*** |  |  |  |  |  |  |  |
| Cluster 1 | Thalamus* | 0 | -22.5 | 1.5 | -12.85 | 254 | 11865 |
| Cluster 1 | Hippocampus_R | 13.5 | -6 | -16.5 | -11.97 | 178 | 11865 |
| Cluster 1 | ParaHippocampal_L | -15 | 0 | -18 | -11.11 | 106 | 11865 |
| Cluster 1 | ParaHippocampal_R | 16.5 | 0 | -16.5 | -10.64 | 170 | 11865 |
| Cluster 1 | Frontal_Sup_Medial_L | 0 | 39 | 28.5 | -10.38 | 658 | 11865 |
| Cluster 1 | Insula_R | 46.5 | 1.5 | 1.5 | -10.36 | 1073 | 11865 |
| Cluster 1 | Hippocampus_L | -12 | -7.5 | -19.5 | -10.15 | 171 | 11865 |
| Cluster 1 | Temporal_Pole_Sup_L | -46.5 | 4.5 | -1.5 | -10.00 | 515 | 11865 |
| Cluster 2 | Calcarine_L* | 0 | -58.5 | 10.5 | -9.06 | 97 | 345 |
| Cluster 2 | Vermis_4_5 | -1.5 | -63 | 0 | -7.13 | 54 | 345 |

**Note:** All patients were categorized into four subgroups according to the PSYRATS AHS score (stage 1/2/3/4=14-24/ 25-29/ 30-32/ 33-42). Results were based on the analyses via spm12 toolbox. Abbreviations of brain regions were according to the AAL template. L, left; R, right. *, peak region of a cluster.

**Supplementary Table 3.** Demographic and clinical characteristic of pAH subgroups divided according to the PANSS P3 score

|  | **Stage 1** (n=19) | **Stage 2** (n=43) | **Stage 3**  (n=25) | **HC** (n=83) | **Statistic** | ***p*** |
| --- | --- | --- | --- | --- | --- | --- |
| Age (year) | 24.95±6.00 | 25.58±5.38 | 24.88±5.80 | 26.80±5.91 | 1.37 | 0.34 |
| Sex (M/F) | 10/9 | 23/20 | 7/18 | 40/43 | 4.64 | 0.20 |
| Education (year) | 12.00±3.16 | 11.43±2.95 | 11.92±3.44 | 14.43±2.65a,b,c | 10.38 | **<0.001** |
| Onset age (year) | 18.26±3.89 | 18.02±4.67 | 19.36±5.92 |  | 0.60 | 0.55 |
| Disease duration  (year) | 6.89±4.38 | 7.58±4.79 | 5.56±3.51 |  | 1.69 | 0.19 |
| First treatment age (year) | 18.95±4.40 | 18.72±5.11 | 20.04±6.02 |  | 0.52 | 0.60 |
| CPZ (mg/d) | 703.77±362.23 | 678.61±273.15 | 630.40±372.65 |  | 0.21 | 0.89 |
| PANSS P3 | 4.00±0.00 | 5.00±0.00a | 6.04±0.20a,b |  | 1989.61 | **<0.001** |
| PANSS total | 53.16±12.02 | 57.00±10.93 | 67.24±15.18a,b |  | 8.01 | **<0.001** |
| PSYRATS AHS | 26.63±5.71 | 27.79±6.28 | 30.84±5.23 |  | 3.25 | **0.04** |
| PSYRATS total | 41.63±8.70 | 37.09±11.59 | 43.72±10.03b |  | 3.39 | **0.04** |

**Note:** Patients scored 4 on the PANSS P3 at stage 1, 5 at stage 2, and 6 or 7 at stage 3 (only one participant scored 7). pAH, schizophrenia with persistent auditory hallucinations; HC, healthy control; M, male; F, female; CPZ, Chlorpromazine equivalent dosage; PANSS, the Positive and Negative Syndrome Scale; PSYRATS, the Psychotic Symptom Rating Scales; AHS, the auditory hallucinations subscale of PSYRATS. The statistic values were obtained by chi-square test or ANOVA. a, *p*<0.05 compared to patients at stage 1; b, *p*<0.05 compared to patients at stage 2; c, *p*<0.05 compared to patients at stage 3.

**Supplementary Table 4.** Results of group comparison of GMV between the pAH subgroups and HC group divided according to the PANSS P3 score

|  |  | MNI coordinate | | |  | Voxel | |
| --- | --- | --- | --- | --- | --- | --- | --- |
| Cluster | Region | x | y | z | *t* value | region | cluster |
| ***Stage 1*** |  |  |  |  |  |  |  |
| Cluster 1 | Thalamus* | 0 | -22.5 | 1.5 | -13.38 | 47 | 4839 |
| Cluster 1 | ParaHippocampal_R | 13.5 | -4.5 | -18 | -13.07 | 193 | 4839 |
| Cluster 1 | Hippocampus_R | 22.5 | -19.5 | -13.5 | -12.94 | 268 | 4839 |
| Cluster 1 | Hippocampus_L | -12 | -7.5 | -19.5 | -10.95 | 248 | 4839 |
| Cluster 2 | Frontal_Sup_Medial_L* | 0 | 34.5 | 34.5 | -10.79 | 634 | 2185 |
| Cluster 2 | ACC_pre_L | 0 | 48 | 4.5 | -9.30 | 333 | 2185 |
| Cluster 2 | Frontal_Sup_Medial_R | 3 | 52.5 | 21 | -8.41 | 402 | 2185 |
| Cluster 2 | Frontal_Med_Orb_R | 3 | 55.5 | 0 | -7.71 | 128 | 2185 |
| Cluster 3 | Insula_L* | -46.5 | 0 | 1.5 | -8.82 | 222 | 626 |
| Cluster 3 | Rolandic_Oper_L | -45 | -9 | 1.5 | -8.44 | 138 | 626 |
| Cluster 3 | Frontal_Inf_Orb_2_L | -39 | 18 | -10.5 | -7.44 | 37 | 626 |
| Cluster 3 | Temporal_Pole_Sup_L | -46.5 | 9 | -3 | -7.04 | 54 | 626 |
| Cluster 3 | Frontal_Inf_Tri_L | -51 | 16.5 | 1.5 | -6.53 | 12 | 626 |
| Cluster 4 | Temporal_Pole_Sup_L* | -42 | 15 | -18 | -8.13 | 290 | 301 |
| Cluster 5 | OFCant_R* | 34.5 | 39 | -19.5 | -7.54 | 82 | 106 |
| ***Stage 2*** |  |  |  |  | 0.00 |  |  |
| Cluster 1 | Thalamus* | 0 | -22.5 | 1.5 | -17.37 | 122 | 8606 |
| Cluster 1 | Hippocampus_R | 15 | -1.5 | -16.5 | -16.19 | 325 | 8606 |
| Cluster 1 | Hippocampus_L | -13.5 | -3 | -16.5 | -14.28 | 295 | 8606 |
| Cluster 1 | ParaHippocampal_L | -12 | -6 | -19.5 | -13.38 | 140 | 8606 |
| Cluster 2 | Frontal_Sup_Medial_L* | 0 | 39 | 30 | -12.10 | 829 | 3509 |
| Cluster 2 | Cingulum_Ant_R | 1.5 | 37.5 | 21 | -9.91 | 399 | 3509 |
| Cluster 2 | Frontal_Sup_Medial_R | 3 | 55.5 | 13.5 | -9.68 | 654 | 3509 |
| Cluster 2 | Cingulate_Mid_R | 1.5 | 3 | 28.5 | -9.25 | 186 | 3509 |
| Cluster 2 | Frontal_Med_Orb_L | 1.5 | 57 | -10.5 | -9.22 | 151 | 3509 |
| Cluster 2 | Frontal_Med_Orb_R | 3 | 64.5 | -9 | -8.50 | 242 | 3509 |
| Cluster 3 | Temporal_Sup_L* | -48 | 0 | 0 | -12.00 | 552 | 2901 |
| Cluster 3 | Temporal_Pole_Sup_L | -51 | 10.5 | -3 | -11.25 | 539 | 2901 |
| Cluster 3 | Frontal_Inf_Orb_2_L | -42 | 18 | -6 | -10.99 | 110 | 2901 |
| Cluster 3 | Rolandic_Oper_L | -45 | -7.5 | 3 | -10.92 | 341 | 2901 |
| Cluster 3 | Insula_L | -31.5 | -3 | 13.5 | -9.67 | 886 | 2901 |
| Cluster 3 | Frontal_Inf_Tri_L | -51 | 16.5 | 0 | -9.64 | 106 | 2901 |
| Cluster 4 | Insula_R* | 33 | -3 | 13.5 | -11.05 | 179 | 286 |
| Cluster 4 | Putamen_R | 30 | 9 | 12 | -6.41 | 21 | 286 |
| Cluster 5 | Cerebellum_4_5_L* | -25.5 | -27 | -31.5 | -10.57 | 82 | 115 |
| Cluster 5 | Fusiform_L | -15 | -31.5 | -18 | -7.48 | 12 | 115 |
| Cluster 5 | ParaHippocampal_L | -16.5 | -28.5 | -21 | -6.92 | 8 | 115 |
| Cluster 6 | Precuneus_R* | 1.5 | -58.5 | 55.5 | -7.88 | 90 | 158 |
| Cluster 6 | Cingulate_Mid_L | 0 | -43.5 | 42 | -5.75 | 12 | 158 |
| Cluster 7 | Frontal_Sup_2_R* | 27 | 21 | 55.5 | -7.79 | 128 | 135 |
| Cluster 8 | Frontal_Sup_2_L* | -24 | 55.5 | 22.5 | -7.50 | 77 | 208 |
| Cluster 8 | Frontal_Mid_L | -27 | 46.5 | 30 | -7.46 | 131 | 208 |
| Cluster 9 | Frontal_Sup_2_R* | 30 | 61.5 | 4.5 | -7.19 | 110 | 116 |
| Cluster 10 | Cerebellum_Crus1_L* | -46.5 | -64.5 | -22.5 | -6.79 | 120 | 146 |
| ***Stage 3*** |  |  |  |  | 0.00 |  |  |
| Cluster 1 | Thalamus* | 0 | -22.5 | 1.5 | -14.09 | 108 | 15010 |
| Cluster 1 | Hippocampus_R | 22.5 | -21 | -13.5 | -12.35 | 270 | 15010 |
| Cluster 1 | Frontal_Sup_Medial_L | 1.5 | 33 | 37.5 | -11.54 | 819 | 15010 |
| Cluster 1 | Calcarine_L | -3 | -57 | 6 | -10.96 | 180 | 15010 |
| Cluster 1 | Hippocampus_L | -21 | -21 | -13.5 | -10.88 | 301 | 15010 |
| Cluster 1 | Calcarine_R | 1.5 | -58.5 | 10.5 | -10.84 | 173 | 15010 |
| Cluster 2 | Frontal_Inf_Orb_2_L* | -42 | 19.5 | -6 | -10.68 | 278 | 3351 |
| Cluster 2 | Insula_L | -45 | 4.5 | -1.5 | -10.37 | 1321 | 3351 |
| Cluster 2 | Rolandic_Oper_L | -45 | -9 | 1.5 | -10.12 | 315 | 3351 |
| Cluster 2 | Temporal_Sup_L | -49.5 | 3 | -1.5 | -9.72 | 402 | 3351 |
| Cluster 2 | Temporal_Pole_Sup_L | -52.5 | 10.5 | -1.5 | -9.17 | 478 | 3351 |
| Cluster 2 | Frontal_Inf_Tri_L | -51 | 18 | 1.5 | -9.08 | 172 | 3351 |
| Cluster 3 | Vermis_3* | 0 | -39 | -10.5 | -9.41 | 72 | 104 |
| Cluster 4 | Frontal_Sup_2_L* | -27 | 58.5 | 9 | -8.19 | 129 | 142 |
| Cluster 5 | OFCpost_L* | -36 | 34.5 | -19.5 | -7.83 | 54 | 159 |
| Cluster 6 | Precuneus_R* | 1.5 | -51 | 48 | -7.69 | 63 | 127 |
| Cluster 7 | Cuneus_L* | -1.5 | -75 | 33 | -6.94 | 106 | 178 |
| Cluster 8 | Frontal_Mid_2_L* | -27 | 46.5 | 30 | -6.63 | 72 | 101 |
| Cluster 9 | Frontal_Mid_2_R* | 30 | 54 | 25.5 | -6.62 | 54 | 104 |

**Note:** All patients were categorized into three subgroups according to the PANSS P3 score (stage 1/2/3=4/ 5/ 6-7). Only one participant scored 7 on the P3 item. Results were based on the analyses via spm12 toolbox. Abbreviations of brain regions were according to the AAL template. L, left; R, right. *, peak region of a cluster.

**Supplementary Table 5.** Causal effect of regions from ROI-wise causal structural covariance network

| Region | MNI coordinate | | | Out-degree | In-degree | Net-degree |
| --- | --- | --- | --- | --- | --- | --- |
|  | x | y | z |  |  |  |
| Thalamus_L | 0 | -23 | 2 | 12.54 | - | 12.54 |
| Frontal_Inf_Oper_R | 48 | 15 | 0 | 1.16 | - | 1.16 |
| Postcentral_L | -49.5 | -16.5 | 46.5 | 1.34 | 0.52 | 0.81 |
| Postcentral_R | 31.5 | -36 | 46.5 | 0.61 | - | 0.61 |
| Thalamus_L | -6 | -22.5 | 4.5 | 1.43 | 0.85 | 0.57 |
| Frontal_Mid_R | 40.5 | 45 | 22.5 | 0.65 | 0.53 | 0.12 |
| Temporal_Sup_L | -63 | -19.5 | 1.5 | 0.33 | 0.56 | -0.23 |
| Rolandic_Oper_R | 57 | 3 | 15 | 0.30 | 0.63 | -0.33 |
| Cingulum_Mid_R | 9 | -6 | 42 | - | 0.52 | -0.52 |
| Occipital_Inf_L | -27 | -91.5 | -6 | - | 0.52 | -0.52 |
| Temporal_Mid_R | 46.5 | -52.5 | 7.5 | - | 0.55 | -0.55 |
| Occipital_Mid_R | 39 | -73.5 | 13.5 | - | 0.55 | -0.55 |
| Supramarginal_L | -49.5 | -25.5 | 22.5 | - | 0.55 | -0.55 |
| Calcarine_R | 19.5 | -69 | 9 | - | 0.59 | -0.59 |
| Postcentral_R | 60 | -10.5 | 39 | - | 0.61 | -0.61 |
| Occipital_Mid_L | -37.5 | -84 | 19.5 | - | 0.85 | -0.85 |
| Cingulum_Ant_R | 7.5 | 43.5 | 22.5 | - | 1.07 | -1.07 |
| Frontal_Inf_Tri_L | -45 | 16.5 | 6 | - | 1.08 | -1.08 |
| Precuneus_R | 15 | -61.5 | 37.5 | - | 1.22 | -1.22 |
| Fusiform_R | 42 | -15 | -28.5 | - | 1.32 | -1.32 |
| Cingulum_Ant_L | -9 | 33 | 21 | - | 1.46 | -1.46 |
| Cerebelum_8_L | -39 | -51 | -49.5 | - | 1.67 | -1.67 |
| Cerebelum_Crus2_R | 7.5 | -85.5 | -30 | - | 2.70 | -2.70 |
| Total |  |  |  | 18.35 | 18.35 | 0.00 |

**Note：**Abbreviations of brain regions were according to the AAL template. L, left; R, right. GC values were transformed by z-distribution, with a *p*<0.05 representing a significant GC value

**

**

**Supplementary Figure 1**. Gray matter volume reduction of the thalamus in the pAH group at stage 1 when sub-grouped according to the PSYRATS AHS score. The GMV reduction in the peak region was still significant after setting the threshold to FWE corrected *p*<10^-9^.
